# Elimination Therapy for the Endemic Malarias

**DOI:** 10.1007/s11908-012-0250-z

**Published:** 2012-03-14

**Authors:** J. Kevin Baird

**Affiliations:** 1 Eijkman-Oxford Clinical Research Unit, Jalan Diponegoro No.69, Jakarta, 10430 Indonesia; 2 Centre for Tropical Medicine, Nuffield Department of Medicine, University of Oxford, Oxford, United Kingdom

**Keywords:** Malaria, Relapse, Primaquine, G6PD deficiency, Radical cure, Gametocytocidal

## Abstract

Most malaria diagnosed outside endemic zones occurs in patients experiencing the consequences of what was likely a single infectious bite by an anopheline mosquito. A single species of parasite is nearly always involved and expert opinion on malaria chemotherapy uniformly prescribes species- and stage-specific treatments. However the vast majority of people experiencing malaria, those resident in endemic zones, do so repeatedly and very often with the involvement of two or more species and stages of parasite. Silent forms of these infections—asymptomatic and beyond the reach of diagnostics—may accumulate to form substantial and unchallenged reservoirs of infection. In such settings treating only the species and stage of malaria revealed by diagnosis and not others may not be sensible or appropriate. Developing therapeutic strategies that address all species and stages independently of diagnostic evidence may substantially improve the effectiveness of the control and elimination of endemic malaria.

## Introduction

Infection of humans by sporozoan parasites of the genus *Plasmodium* causes malaria. That term describes a diverse set of diseases provoked by at least five species (Table 1) passing through complex life cycles of morphologically and physiologically distinctive forms, each of which manifests clinical consequences ranging from none to death. Protean life threatening syndromes occur among the species at rates ranging from often to very rare: coma, severe anemia, respiratory distress, renal or hepatic failure, shock, splenic infarct or rupture, and splenomegaly. Specific forms of parasites appear only within a particular host (human or mosquito) and organ (human liver or blood), and each exhibits unique susceptibilities to various chemotherapeutic agents [1]. A drug that kills one stage typically has little effect on other stages. Some drugs may kill a particular stage of one species but not the same stage of another (Table 2). Species- and stage-specific resistance to drugs varying geographically greatly deepens the complexity of the chemotherapeutic problem. Rational strategy for the chemotherapeutic management of patients with malaria thus requires expert biological, pharmacological, epidemiological, and clinical considerations.

**Table 1:** The malarias of humans

	*P. falciparum*	*P. vivax*	*P. malariae*	*P. ovale*	*P. knowlesi*
Epidemiology	Endemic/Epidemic; pan-tropical excluding South Pacific east of Braxton Line	Endemic/Epidemic; pan tropical excluding South Pacific east of Braxton Line, and sub-tropical and temperate	Endemic but highly focal; pan-tropical excluding South Pacific east of Braxton Line	Endemic in Africa; endemic at very low frequencies in South Asia, Southeast Asia, and Oceania west of Braxton Line	Zoonosis from macaques native to Southeast Asia; forested areas only
Consequence	Very often life-threatening	Often life-threatening	Rarely life-threatening	Rarely life-threatening	Often life-threatening
Threatening syndromes	Anemia, cerebral, hyper-parasitemia, pulmonary, renal, hepatic, shock	Anemia, cerebral, pulmonary, renal, hepatic, hemorrhage, shock, splenic rupture	Renal, splenomegaly	Pulmonary, shock	Cerebral, pulmonary, renal, hepatic, shock
Chronic sub-patency or latency forms	Asexual & sexual blood stages in the semi-immune	Asexual & sexual blood stages in the semi-immune; dormant stage in liver up to 3 years	Asexual & sexual blood stages in any patient, very long term latency of several decades	Asexual and sexual blood stages in the semi-immune; dormant stage in liver	None known
Diagnostic blind spots	Parasitemia <200/μL (RDT); <20/μL (expert microscopy); <1/μL (PCR)	Parasitemia <500/μL (RDT); <20/μL (expert microscopy); <1/μL (PCR); dormant liver stage; bone marrow (?)	Parasitemia <200/μL (RDT); <20/μL (expert microscopy); <1/μL (PCR)	Parasitemia <500/μL (RDT); <20/μL (expert microscopy); <1/μL (PCR); dormant liver stages	No RDT; \<20/μL (expert microscopy); <1/μL (PCR)
Radical cure	Blood schizontocide(s) + gametocytocide	Blood schizontocide(s) + hypnozoitocide (gametocytocide)	Blood schizontocide(s) + gametocytocide	Blood schizontocide + hypnozoitocide (gametocytocide)	Blood schizontocide + gametocytocide

**Table 2:**
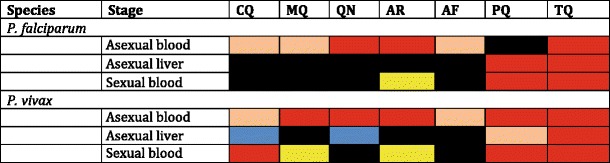
Mosaic of the effects of some antimalarials at therapeutic doses against stages of *P. falciparum* and *P. vivax* malarias

***AF*** antifolates; ***AR*** artemisinins; ***CQ*** chloroquine; ***MQ*** mefloquine; ***PQ*** primaquine; ***QN*** quinine; ***TQ*** Tafenoquine

Red = effective; Pink = effective but with resistance in some strains; Yellow = limited effect; Black = no effect; Blue = effective but only in combination with primaquine (the only drugs now known to synergize primaquine and tafenoquine activity against hypnozoites)

Malaria treatment guidelines from authoritative agencies invariably list recommendations according to species of parasite and stage targeted [2, 3]. Malariologists and healthcare providers alike maintain this segregation, as do developers of those chemotherapies. Species- and stage-specific treatment guided by a confirmed diagnosis is the keystone of malaria chemotherapeutics strategy and practice. There is obvious appeal in this approach, i.e., mitigating the daunting complexity of the malaria chemotherapeutics problem to manageability in practice [4]. This may be especially practical in non-endemic zones where malaria patients typically have access to state-of-the-art diagnostics and present with a single species diagnosis. In contrast, patients in endemic zones often carry two or more species and stages of parasite out of diagnostic reach for technical or practical reasons. Directing therapy only against that which diagnosis affirms may not be sensible—it may virtually ensure incomplete and ineffective chemotherapeutics in the context of control or elimination of the endemic malarias. Should malaria treatment in endemic zones be separated, in both a strategic and material sense, from that for patients elsewhere? This review examines evidence on this important question.

## Northern Chemotherapeutics Bias

Resource-rich and endemic malaria-free nations of the Northern hemisphere set the malaria chemotherapy agenda during the 20th Century and continue doing so [5]. Chemotherapeutics strategies and resources have focused almost exclusively on the acute attack of falciparum malaria. This carries important ramifications for endemic nations, i.e., neglect of chemotherapeutic attack of the many other malarias. The blurring of chemotherapeutics strategies aimed at travelers versus residents sharply inhibits development of therapies relevant to malaria control and elimination. Treatment as a key element of a larger malaria control strategy that includes rational attack on all of the endemic malarias has been grossly neglected [6].

Many drugs are available for one stage and species—the disease-causing asexual blood stages of *Plasmodium falciparum*. This has long been, and continues to be, the chemotherapeutic imperative of antimalarial drug developers. These therapies may also be applied to cure acute attacks of other species, but recommendations for such rarely come with adequate evidence of safety and efficacy, much less optimized dosing. The non-falciparum acute malarias have long been viewed as far less threatening to life and thus relatively unimportant [7, 8]. The zoonosis of *Plasmodium knowlesi* is certainly threatening, but relatively infrequent. The view of vivax malaria as a benign infection has been recently challenged with evidence of substantial burdens of morbidity and mortality in endemic zones [9–12, 13••]. The misperception of *Plasmodium vivax* as a benign entity largely explains the neglect of therapies aimed at its dormant liver stage, the hypnozoites causing relapses—*P. falciparum* does not form hypnozoites and has no known chronic latent stage. Gametocytes of any species pose no direct clinical threat to patients and their treatment has been likewise neglected.

The only drug with a licensed indication for therapy aimed at hypnozoites or gametocytes is primaquine. That drug causes mild to severe hemolysis in patients having glucose-6-phosphate dehydrogenase deficiency (G6PDd), an inborn and typically silent disorder most common in malaria endemic zones [14]. The exclusion of such patients from risky primaquine treatments rarely occurs in endemic zones for lack of laboratory capacities where most malaria patients live. These providers rarely prescribe a potentially threatening therapy against an infection perceived (inappropriately) as non-threatening. Regimens of primaquine therapy demonstrated to be safe among the most sensitive variants of G6PDd, and thus useful without the necessity of screening, have not been adequately explored. Consequently, despite this drug being potentially extremely useful in controlling malaria, it remains rarely used in endemic zones and thus largely ineffective in a global malaria sense [15•].

Primaquine in the hands of providers able to routinely and safely exclude G6PDd patients is very often prescribed (>87% [16]). Primaquine was developed and optimized for use by the US military, and it has effectively served that and similar populations for over five decades. Its impracticality and ineffectiveness in endemic zones largely escaped notice and solutions to the problem, e.g., universally safe dosing or a simple point-of-care G6PDd diagnostic device, have not emerged. The chemotherapeutic requirements of developed nations had been met and exploration of safer options to standard primaquine therapy has not been a research priority. An experimental drug in phase III trials in 2011 and intended to replace primaquine, tafenoquine (GlaxoSmithKline, UK), presents many of the same limitations with respect to safe use in endemic zones, i.e., hemolytic toxicity among G6PDd patients. The utility of that drug in most malaria patients may hinge upon the availability of safe dosing or G6PDd diagnostics suited for practical use at the point-of-care typical of endemic zones.

Northern chemotherapeutics has narrowly focused on the acute attack of falciparum malaria to the exclusion of other avenues of chemotherapeutics development vital to controlling and eliminating the endemic malarias. This neglect resulted in a single therapeutic option for most other malarias—primaquine, a 60-year old drug that cannot be safely applied in endemic zones. Addressing this very broad problem requires deeper appreciation of the nature of the entrenched endemic malarias and the need for therapies aimed at them rather than solely against that which diagnosis affirms.

## Non-Endemic vs. Endemic Chemotherapeutic Practice

Among residents of the developed world, travelers represent the primary population affected by malaria. Although outbreaks of locally acquired malaria sporadically occur, especially in the United States, the vast majority of patients with malaria in North America, Europe, and Japan are travelers who acquire their infections abroad. In the USA in 2009, for example, 1,478 of 1,484 (99.6%) confirmed malaria cases were classified as imported; and the remaining 6 patients were attributed to transfusion, transplant, or possible congenital transmission [17•]. Typically more than 98% of malaria diagnosed in travelers is limited to a single species [17•, 18–20]. A species-specific diagnosis is often rendered using molecular diagnostics or reference laboratory microscopy, and that diagnosis guides appropriate therapy.

In this setting, malaria is an isolated and relatively rare clinical problem rather than a broad public health concern. As such its management is in the hands of providers who may have little experience with the disease but who typically have access to state-of-the-art clinical and laboratory capacities. The treatment of malaria in travelers thus emphasizes species-specific therapies in individual patients, guided by appropriate laboratory evidence and screening against possible contraindications among therapeutic options, leading to a very high probability of complete recovery. The case fatality rate for malaria in the USA in 2009, for example, was 0.3% [17•].

In contrast, chemotherapeutic tools, policy and practices in endemic zones aim at whole populations at risk rather than isolated patients. The provider of treatment is very often the patient himself or a neighbor with specialized and limited training [21, 22•]. Management of malaria by physicians or nurses in a clinic or hospital often represents a last resort following the failure of more convenient and less costly approaches, even in relatively large cities [23]. In most endemic zones treatment of malaria often comes without a laboratory or rapid diagnostic confirmation of malaria [21–27], much less screening for contraindications for any given therapy.

As the example of primaquine starkly illustrates, therapies suited to healthcare in the developed world may prove inadequate to healthcare delivery in the developing world. The developing world requires chemotherapeutics that may be safely and effectively administered without clinical supervision and risk of dangerous complications. Further, treatment of malaria very often occurs without a species diagnosis. Development of therapies suited to safe and effective use in advanced healthcare systems may contribute little to mitigating malaria as a global problem overwhelmingly borne by the most economically disadvantaged people. Chemotherapeutic options for them must be safe and practical with little clinical and laboratory capacities.

## Non-Endemic vs. Endemic Risks

Other important factors distinguish malaria as a narrow clinical versus broad public health problem. Endemic malaria encompasses populations hosting all of the malarias, whether patent, sub-patent, or dormant/latent (Table 1). The febrile patient with patent parasitemia seeking treatment likely represents a relatively rare occurrence offering an opportunity for diagnosis and specific therapy. Most of the malarias in endemic communities occurring below diagnostic thresholds or capacity (Table 1) supports that likelihood. Evidence also demonstrates that even a febrile patient successfully diagnosed and treated for one species and stage of parasite, very often carries others. These may be dormant hypnozoites (for *P. vivax* and *P. ovale*) or low-grade and asymptomatic asexual and sexual parasitemia (all species). Chemotherapeutic attack on only the species confirmed by diagnosis likely incurs substantial clinical and public health risks. This may be most plainly illustrated by the failure to attack hypnozoites with primaquine even with a diagnosis of *P. vivax,* as commonly occurs in endemic zones.

## Hypnozoite Reservoir

**Fig. 1:**
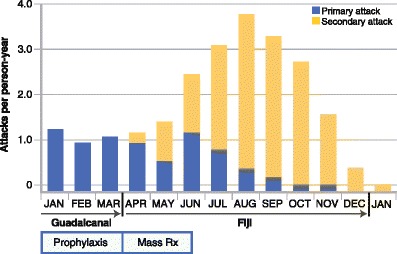
Graph illustrates malaria attack rates among the U.S. Army Americal Division at Guadalcanal and malaria transmission-free Fiji during 1943 (adapted from [28]). The attacks of Fiji represent the burden of disease imposed by untreated relapse of vivax malaria

The experience of the U.S. Army Americal Division at the battle for Guadalcanal in the Solomon Islands during 1942–1943 illustrates the vitally important public health consequences of an unchallenged hypnozoite reservoir of infection (Fig. 1). During January through March 1943 these soldiers fought on that highly malarious island and were provided atabrine (also called mepacrine or quinacrine) for suppressive (blood schizonticidal) chemoprophylaxis. They nonetheless suffered malaria attack rates of 1.7/person-year. Prior to their evacuation from Guadalcanal for rest and recuperation in non-malarious Fiji—anopheline mosquitoes do not occur east of the Braxton line in the South Pacific—the soldiers received therapeutic doses of atabrine en masse. The malaria attack rates on Fiji in this army division, peaking at 3.7/person-year in August 1943, was all *P. vivax* and, given the absence of anopheline mosquitoes, almost certainly derived from latent hypnozoites [28]. These attack rates provide a glimpse at possibly heavy public health consequences attending failure to consider and treat silent malarias like latent *P. vivax*.

Modern studies affirm such concerns. Douglas et al. [29••] conducted an exhaustive review of 10,549 study subjects with naturally acquired (in Thailand and neighboring nations) acute *P. falciparum* malaria treated with standard or experimental therapies for that attack. After 63 days of observation with little risk of reinfection, 51% of subjects (receiving rapidly excreted blood schizonticidal therapies that would not interfere with relapses) had experienced an attack of *P. vivax* malaria. That brief period of follow-up may have missed many subsequent attacks (see Figure) and the 51% rate may thus be considered a highly conservative estimate of the prevalence of latent *P. vivax* among patients with acute falciparum malaria in the Mekong region. Many prior studies from that region reported essentially similar findings [30–32], [33•]. The report of Douglas et al. [29••] effectively removes doubt regarding a very high prevalence of one of the silent malarias (the evidence speaks only to hypnozoites of *P. vivax*) despite a setting that is considered relatively low transmission intensity. Hypnozoites in most endemic zones evidently pose a serious threat to public health. And yet the only therapy against this malaria, primaquine, cannot be applied due to risk of harm to G6PDd patients and the inability to effectively and safely exclude them from treatment.

## Mixed Infections

Recent studies also point to blood stage infections in endemic zones as often being a mix of species. The diagnosis of mixed species infection by microscopy is notoriously difficult and insensitive: the parasitemia tends to be dominated by one species and microscopists often fail to spot the minority species. Molecular diagnostics usually detect several-fold higher proportions of mixed infections relative to standard microscopy. Nested PCR techniques applied to blood samples from Thailand, for example, found 23% to 24% mixed infections near the Myanmar border, and 3% to 5% in eastern and southern areas of that nation [34]. PCR-based work in nearby Cambodia showed mixed infection rates of 23% around Rattanakiri in the northeast [35•]. Mixed infection rates among positives were 6.5% among samples from Afghanistan, and 22% and 24% among samples from Iran and Pakistan [36]. A large sample of 2527 residents of hyper- to holo-endemic Papua New Guinea was analyzed using a multiplex molecular methodology (ligase detection reaction—fluorescent microsphere assay): among the 1844 (73%) positive for any species of malaria, 61% were positive for a single species of parasite. Among the others, 30%, 8%, and 1% were positive for 2, 3, or 4 species, respectively [37••]. Mixed species infection of blood thus typically occurs in about 25% of patients with malaria in the Asia-Pacific region, and ranges between about 5% and 40%. And yet treatment aims only at that which diagnosis affirms.

## Asymptomatic Parasitemia

The asymptomatic carrier state of blood infection represents another important risk in endemic zones. Despite the long-held conviction that effective immunity to febrile illness by the plasmodia requires chronic and heavy exposure to infection [38, 39], recent evidence casts doubt. In 2008 a mass blood survey of 9491 residents of Temotu Province, Solomon Islands, for malaria by microscopy experts revealed only 256 positive patients (2.7%) and only 18% of these were febrile [40••]. *Plasmodium vivax* dominated at 82% of infections, and most of these (66%) were below 100 parasites per microliter (40% of *P. falciparum* were below that threshold). Examination by PCR diagnostics showed a prevalence of 9%, suggesting that <30% of active blood infections were detected by expert microscopy, and that the rate of fever with blood infection may have been closer to just 5% despite very low transmission intensity. Essentially similar findings have been reported from Amazonia [41, 42•], Africa [43], the Middle East [44], and Southeast Asia [45, 46]. Most people with malaria in endemic zones will not be suffering illness and will harbor blood stage parasites below the detection thresholds of even expert microscopy, and very far below the widely available rapid diagnostic (RDT) kits (considered reliable only with >200 parasites/μL [47]). The reality of an apparently large reservoir of blood infection beyond diagnostic reach impels consideration of mass drug administration in the context of elimination strategies [48].

## Malaria of Travelers Versus Residents

The silent malarias represent the plasmodia provoking no symptoms over an extended period and occurring beyond the reach of available diagnostics. That silence may be a consequence of biology (hypnozoites & gametocytes do not cause illness at any density), technology inadequate to diagnostic confirmation at a given density, lack of access to a diagnosis of any sensitivity, or a semi-immune host oblivious to carrying the infection. Except for the threat of relapse in travelers, these silent malarias do not register as a chemotherapeutics problem in non-endemic settings. As contemporary experimental challenge studies demonstrate, non-immune travelers become acutely ill at very low parasite densities compared to the chronically exposed [49, 50]. Passive detection and diagnosis of malaria in non-endemic zones probably captures nearly all infections, but very few in endemic zones.

Acknowledging endemic malaria as distinct from the strictly clinical problem of non-endemic malaria begins to address the extremely poor fit of current antimalarial chemotherapeutics development and practice to malaria as it occurs in the endemic world. The points defined in Table 3 highlight key distinctions between these two worlds of malaria.

**Table 3:** Non-Endemic versus Endemic Malarias

Rare versus routine	Large developed countries see many hundreds or a few thousand cases of malaria each year; whereas large undeveloped nations like India or Indonesia likely experience at least several million cases each.
Resource rich versus resource poor	Almost all malaria cases in the developed world will be managed by physicians, nurses, and laboratorians supported by state-of-the-art clinical and laboratory facilities; whereas most cases in the developing world will likely be self-treated or treated by a neighbor with specific training.
Single versus multiple	Malaria in travelers will often carry a single confirmed species; whereas residents of endemic zones will be much more likely to carry an accumulation of silent species and stages beyond diagnostic reach for technical or practical reasons.
Symptomatic versus asymptomatic	Most patients in the developed world will experience clinical malaria even with very low parasitemia; whereas most residents of endemic zones will carry asymptomatic infections in their bloodstream and liver.
Robust versus vulnerable	Travel abroad typically precludes the most vulnerable, and travelers thus represent the most robust in both a financial and health sense; whereas people residing in endemic zones often represent the most vulnerable in both a financial and health sense.

Taken together these distinctions effectively define two separate malaria problems: non-endemic versus endemic, or the malaria of travelers versus residents. The importance of this distinction was made as early as 1902 by Sir Ronald Ross and is periodically re-emphasized [51]. Nonetheless, the blurring of these two problems in a chemotherapeutics sense continues to sharply inhibit development of strategies and tools suitable for practical application in the endemic world.

## Endemic Chemotherapeutics Strategy

Greater awareness of the nature of endemic malaria would likely permit development of more effective strategies for attacking the silent malarias that dominate those zones. The long obsession with the acute attack of falciparum malaria, although obviously a very important problem in any setting, attended the frank neglect of other important avenues of chemotherapeutics research and development. The sole availability of primaquine against hypnozoites and gametocytes, a drug that cannot today be safely used in endemic zones despite six decades of availability, affirms this neglect [15•]. Drug discovery should include new tools aimed specifically at those malarias [52]. In the meantime chemotherapeutics and control strategists should consider optimizing use of available drugs for endemic settings.

The reality of treatment without diagnosis in endemic zones, whether by unavailability or inadequate sensitivity, should be accepted. Abandoning diagnosis-driven species-specific therapy as the keystone of malaria chemotherapeutics allows development of treatments intended to provide good efficacy against all species and stages. Primaquine as the only available hypnozoitocide and gametocytocide brings focus to the immediate problem: safety in G6PDd patients. Administering primaquine to all malaria patients, regardless of G6PDd status or diagnostic outcome or availability, may not appear impractical or unreasonable if several avenues of evidence, long overdue, become developed.

That development may be rationally focused on key discreet questions. Applying hypnozoitocidal primaquine therapy would almost certainly provide incidental gametocytocidal coverage. This brings sharp focus to the essential research question: what hypnozoitocidal regimen of primaquine may be safely and effectively administered to patients with any malaria regardless of G6PDd status? The US Army developers of primaquine addressed this question to vivax malaria in the 1950s and derived an answer: a 45 mg weekly dose of primaquine for 8 weeks offered good safety and efficacy against relapse by *P. vivax*. Their answer, however, was not universally applicable because they evaluated only healthy volunteers having the relatively mild African A- variant of G6PDd, and very few of them [15•]. That regimen nonetheless provides a useful starting point for developing a more complete answer to the essential question. Recent work in Pakistan with the 8-week regimen of primaquine demonstrated good safety and efficacy, but only a single G6PDd subject was enrolled [53••]. Is the single weekly 45 mg adult dose safe among G6PDd variants in a particular area of control operations?

An affirmative answer to this question would provide a chemotherapeutic tool of immeasurable value to the malaria control programs responsible for those operations. But making that determination site-by-site and variant-by-variant may be impractical and unnecessary. Instead, a globally relevant answer may be developed that permits treating all malarias with a single primary therapy proven safe and effective even in those with G6PDd of the most sensitive variants. That is the strategic aim. How to realize it?

## Rationale for Elimination Therapy Research and Development

The term radical cure has historically been applied in a species-specific sense in malariology. Radical cure implies killing all infecting stages: all blood stages for the non-relapsing malarias, and all blood and liver stages for the relapsing malarias. In a practical sense, radical cure means administering blood schizontocide with gametocytocide versus blood schizontocide with hypnozoitocide (with incidental gametocytocidal activity) for the non-relapsing and relapsing species, respectively (Table 1). Aiming at all species and stages with a single course of therapy, on the other hand, may be termed elimination therapy.

## Available Tools

The critical need for drug discovery across all of the malarias has been explained, but the focus here is upon research applying drugs available in 2011. Therapies aimed at all of the malarias would include blood schizontocide(s), hypnozoitocide(s), and gametocytocide(s). Primaquine (and perhaps tafenoquine) as the only option for the latter two compartments simplifies the development algorithm, i.e., pair it with an appropriate blood schizontocide(s). This is what the developers of primaquine did with chloroquine and primaquine in fielding the only radical cure for vivax malaria. The added requirement of efficacy against falciparum malaria should be a relatively simple problem given the mostly shared susceptibilities to blood schizonticides by the asexual blood forms (Table 2).

**Fig. 2:**
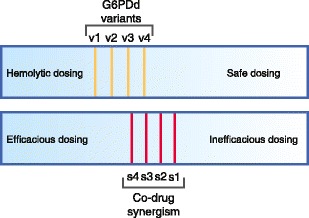
The relationship between safety & efficacy with primaquine among the G6PDd. Hypothetical primaquine dosing (dark = high dose; light = low dose) among G6PDd variants of variable sensitivities (v4 most sensitive) and synergistic effects of other drugs administered with primaquine (s1 most synergistic) against relapse revealing dosing at which universally safe and efficacious primaquine therapy may be possible, i.e., when administered with s1 or s2 effects. Each line represents the division between hemolytic vs. safe (yellow lines), and efficacious vs. inefficacious (red lines) for each given variant or co-drug, respectively

The relatively wide range of therapeutic options for treatment of the asexual blood stages of all species offers flexibility in optimizing the obligatory hypnozoitocidal primaquine (or tafenoquine) partner for efficacy in elimination therapy. Maximizing that efficacy also directly addresses the primary practical concern: increasing safety among many G6PDd variants. This helps define the evidence needed to guide decisions on primaquine dosing. That work must emphasize defining primaquine sensitivity phenotypes among G6PDd variants, thereby informing strategy for minimizing effective hypnozoitocidal/gametocytocidal primaquine doses within range of safe tolerability. Figure 2 visualizes this concept. If a synergistic co-drug (s1) diminishes the minimally effective dose of primaquine to a level below the hemolytic sensitivity of the most sensitive G6PDd variant (v4), a dosing window for universally safe treatment strategy may emerge.

## Minimizing Harm—Synergies

Although not widely acknowledged or understood today, therapeutic activity of primaquine against hypnozoites requires an appropriate companion drug [54]. When administered following rather than concurrently with quinine, for example, primaquine failed against relapse [55]. The same concurrent dose of primaquine proved less effective when administered with chloroquine rather than quinine [55], suggesting chemical class-specific synergistic effects. An exploration of those effects may yield a partner hypnozoitocidal drug (whether blood schizonticidal or not) yielding less threatening therapeutic doses of primaquine. Indeed, recent work with tafenoquine against the *Plasmodium cynomolgi* model of *P. vivax* relapse in rhesus macaques showed a 10-fold reduction in the minimal effective dose of that drug when administered with blood schizontocide [56••]. Likewise, the widely used ACTs show good activity against young but not mature gametocytes; optimizing ACT-primaquine to gametocytocidal activity may also yield less threatening dosing—a strategy that may prove vitally important in Africa where hypnozoitocidal therapies may not be indicated by virtue of the relative paucity of vivax malaria.

## Preventing Harm—G6PDd Screening

The core G6PDd problem with primaquine, and therefore elimination therapy, may also be addressed by a point-of-care diagnostic that is robust in endemic settings and across variants and clinical conditions. The kit must reliably exclude those at risk under almost all circumstances of therapy, including variable genotypes of G6PDd, states of heterozygosity among females (lyonization of this X-linked trait), and states of disease caused by malaria and other infections or nutritional and physiological disorders. This approach, like that of fielding a universally safe regimen of primaquine or tafenoquine, requires certain knowledge of hemolytic sensitivity phenotypes among G6PDd variants. Today only scanty evidence informs that critical knowledge base.

## G6PDd Sensitivity Phenotype as a Core Chemotherapeutics Research Agenda

Development of elimination therapy requires characterizing primaquine sensitivity phenotypes among a range of rationally selected G6PDd variants. Among the many dozens of known variants, primaquine sensitivity phenotype has been characterized for only three: African A-, Mahidol, and Mediterranean B-. These represent mild, moderate, and severe phenotypes, respectively. The residual enzyme activity among these variants happens to inversely correlate with that sensitivity. The same relationship is often presumed to exist among the many other variants [57], but this has not yet been quantitatively demonstrated. At least one case illustrates the uncertainty in this correlation: an Iranian G6PDd patient with 19% residual enzyme activity (approximately that typically observed in mildly primaquine sensitive African A- subjects) suffered a deep hemolysis and required blood transfusion after a single 45 mg dose of primaquine [58]. Research should be aimed at filling important gaps in understanding the mechanism(s) underlying primaquine toxicity process and consequence among G6PDd variants. That ranges from relatively inconsequential to life threatening and the key determinants remain unknown.

Two common variants illustrate the profound differences between them with respect to primaquine sensitivity. Healthy African-American subjects with A- G6PDd experience a relatively mild and self-limiting primaquine-induced hemolysis. As susceptible older red blood cells are replaced, hemolytic sensitivity vanishes. In one clinical trial African A- G6PDd subjects received 30 mg primaquine daily for over 4 months: hematocrit levels troughed at about 30% hemolysis after 10 days and recovered to normal levels within 2 weeks despite continuing exposure to that high dose of primaquine [59]. This self-limiting toxicity contrasts with that in subjects with Mediterranean B- G6PDd. In those subjects even reticulocytes and younger red blood cells replacing hemolyzed red blood cell populations remained exquisitely primaquine-sensitive: hemolysis deepened with each daily dose [60]. The obvious choice of variant for exploring universally tolerable and safe regimens of primaquine would be a variant like Mediterranean B-. Dosing regimens tolerated by these patients would likely also prove tolerable among patients with most other variants (Fig. 2).

Studies of complete G6PD gene sequences among populations of G6PDd variants may reveal even deeper complexity in the primaquine sensitivity problem. Common variants have been conventionally identified by enzyme biochemistry phenotypes (mobility, stability, substrate kinetics) linked to single mutations confirmed by restriction fragment length polymorphisms or sequencing only tiny fragments of the G6PD gene. This very large gene, however, may contain a host of other single nucleotide polymorphisms (SNPs) relevant to primaquine sensitivity phenotype [61]. Complete G6PD gene analyses of variants representing distinct primaquine sensitivity phenotypes may radically improve understanding of essential aspects of the phenomenon.

The essential research and development question, “What hypnozoitocidal regimen of primaquine may be safely administered to patients with any malaria regardless of G6PDd status?” may be focused upon variants of greatest sensitivity to primaquine (per Fig. 2). Available data on primaquine sensitivity in the most-sensitive (thus far known) Mediterranean B- does not preclude the possibility of safe and effective dosing. It is known only that sustained daily dosing with the relatively high 30 mg dose constitutes a potentially lethal threat to such patients [60]. A unique characteristic of the hypnozoitocidal activity of primaquine (called the total dose effect) offers the possibility of safe and efficacious options, i.e., lower doses over longer periods. A single total dose of primaquine, whether administered at once or weekly over many weeks, provided equally efficacious activity against relapse [62, 63]. This unique characteristic was exploited by the developers of primaquine in identifying the 45 mg weekly primaquine dose for 8 weeks. The safety of this regimen in Mediterranean B- or similarly very sensitive variants has not been evaluated. Finally doing so may be the first step in the overdue exploration of elimination therapies.

Establishing the safety of a weekly 45 mg dose for 8 weeks in the most vulnerable G6PDd patients would provide national malaria control programs with the evidence required to assertively and confidently apply primaquine as routine therapy without G6PDd screening. On the other hand, if primaquine at this dose indeed threatens such patients, especially those already acutely ill with malaria, then the necessity of optimized lower doses of primaquine (Fig. 2), or of point-of-care G6PDd screening kit would be appreciated as necessary in developing and fielding elimination therapies. Pursuit of either avenue requires much greater understanding of primaquine sensitivity genotypes and phenotypes among G6PDd variants.

## Critical Questions

Working to develop elimination therapies in the short term requires addressing critical questions regarding the hemolytic toxicity of primaquine and other 8-aminoquinolines among the G6PDd. Although malariologists often cite parasite resistance to drugs as a key determinant of inadequate control of endemic malaria, it seems likely that the long-standing strategy of partial and fragmented chemotherapeutic attack may also explain that problem. The search for solutions to what is perhaps the essence of persistent endemic malaria should focus upon primaquine and tafenoquine safety across important G6PDd variants, and their efficacies across chemotherapeutic compartments.

1. Does G6PDd residual enzyme activity correlate with primaquine sensitivity phenotype among the G6PDd?
2. What G6PDd genotypes are linked to primaquine sensitivity phenotype?
3. What are the most vulnerable G6PDd genotypes and their frequencies and geographic distributions?
4. What is the threshold of exposure to primaquine among most sensitive G6PDd variants that may be considered not threatening to patient safety?
5. Can the hypnozoitocidal and gametocytocidal doses of primaquine and other 8-aminoquinolines be substantially reduced by application with other drugs that maximally synergize that activity, whether blood schizonticidal or not?
6. Can optimized hypnozoitocidal and gametocytocidal therapies be translated into dosing that is safe and effective in the most vulnerable G6PDd variants?
7. Can a point-of-care diagnostic for G6PDd safely exclude patients at risk of harm caused by any regimen of primaquine, and do so at very low cost without the need for a cold chain or laboratory capacities?

## Conclusions

The extraordinary efficacy of primaquine and other 8-aminoquinolines in killing across species and stages of plasmodia has not been capitalized as a result of the hemolytic toxicity of this class of compounds among G6PDd patients. G6PDd and the failure to develop practical solutions to this problem may explain the absence of elimination therapy in the malaria control toolbox. That failure stems from another: little appreciation of the important distinctions between non-endemic and endemic malarias as regards chemotherapeutics strategies and practice. Clinical investigators in endemic zones have raised the issue of elimination therapy for all malarias [64], but the possibility of safe and effective regimens of primaquine combined with blood schizonticides and aimed at elimination therapy remains largely unexplored. Drug development paradigms today remain highly segregated by type of malaria, and largely focused upon blood schizonticides for acute falciparum malaria. Development of drug combinations against all malarias may provide a means of fielding therapies appropriate and effective in endemic settings. Current strategy and practice, well suited to malaria as it occurs in developed nations, leaves most of the malarias in endemic zones unchallenged.
